# Risk of Developing Checkpoint Immune Pneumonitis and Its Effect on Overall Survival in Non-small Cell Lung Cancer Patients Previously Treated With Radiotherapy

**DOI:** 10.3389/fonc.2020.570233

**Published:** 2020-09-29

**Authors:** Feliciano Barrón, Roberto Sánchez, Marisol Arroyo-Hernández, Carolina Blanco, Zyanya L. Zatarain-Barrón, Rodrigo Catalán, Maritza Ramos-Ramírez, Andrés F. Cardona, Diana Flores-Estrada, Oscar Arrieta

**Affiliations:** ^1^Thoracic Oncology Unit, Instituto Nacional de Cancerología, México City, Mexico; ^2^Cancer Center, ABC Medical Center, México City, Mexico; ^3^Clinical and Translational Oncology Group, Clínica del Country, Bogotá, Colombia; ^4^Foundation for Clinical and Applied Cancer Research – FICMAC, Bogotá, Colombia; ^5^Clinical Research and Biology Systems Department, Universidad el Bosque, Bogotá, Colombia

**Keywords:** checkpoint immune therapy, pneumonitis, radiotherapy, NSCLC, lung cancer, immune related adverse effects

## Abstract

**Introduction:**

Immune checkpoint inhibitor-related pneumonitis (ICIP) is a potentially life threatening immune-related adverse event (irAE), especially in non-small cell lung cancer (NSCLC) patients. Currently, the potential for increased irAE in patients who receive radiotherapy is scarcely known, although a connection between antitumor immune responses and irAEs has been suggested. In this study, we evaluated the development of ICIP in non-small cell lung cancer patients with prior radiotherapy, treated with immunotherapy in the second-line.

**Methods:**

In this retrospective trial, we included patients treated with second-line immunotherapy at the National Cancer Institute in Mexico City from February 2015 to February 2018. Clinical, radiological and treatment variables were evaluated according to the presence of ICIP as defined by the Common Terminology Criteria for Adverse Events (4.0) in patients with or without a previous (≥months) history of radiotherapy.

**Results:**

Among 101 NSCLC patients who received treatment with ICIs, 22 patients (21.8%) were diagnosed with ICIP, of which 73% (16/22) had a history of radiotherapy (OR 6.04, 95% CI 2.03−18.0, *p* < 0.001). Median progression free survival and overall survival were similar in patients who developed ICIP compared with those who did not, however, patients who presented grade ≥ 2 ICIP had an increased risk of mortality (HR 2.54, 95% CI 1.20−5.34, *p* = 0.014).

**Conclusion:**

In this real-world cohort of NSCLC patients treated with ICI, the history of prior radiotherapy was associated with increased risk for ICIP development. Unlike other irAEs, grade ≥ 2 ICIP is an independent prognostic factor for decreased survival in NSCLC patients.

## Introduction

Immune-checkpoint inhibitors anti-PD-1 and PD-L1 have changed the paradigm of treatment in several malignancies, including non-small cell lung cancer (NSCLC). Immunotherapy has demonstrated to improve overall survival (OS) and is now the standard of care for many solid tumors. The inhibition of the PD-1/PD-L1 axis can disrupt normal mechanisms of immune tolerance, resulting in increased immune activation in normal tissues; this in turn, can be associated with a unique set of adverse side effects, also known as immune-related adverse events (irAEs). Among them, immune checkpoint inhibitor-related pneumonitis (ICIP) has been reported as a potentially life-threatening irAE (1, 2).

Currently, a considerable subset of patients with locally advanced unresectable or metastatic NSCLC receive treatment with radiotherapy, alone or in combination with chemotherapy before being candidates to receive immunotherapy (3, 4). Moreover, radiotherapy has an important role in patients with brain metastases, as well as in radical treatment of patients with oligometastatic disease (5, 6).

Radiotherapy offers good local control of tumor growth; however, it also has multiple immune-modulatory effects. Radiation therapy induces DNA and membrane cellular damage, increasing reactive oxygen species (ROS) that activate transcription factors and signaling pathways modulating the immunophenotype and immunogenicity of tumor cells, restoring antitumor T-cell response in the tumor microenvironment, increasing tumor antigen release, while also improving antigen presentation and T-cell infiltration (7, 8). New evidence suggests that immunotherapy enhances antitumor immunogenicity of radiotherapy when used after local control with radiation (9). Fractionated radiotherapy in combination with anti PD-1/PD-L1 monoclonal antibodies was shown to generate effective CD8+ T-cell responses that improve local tumor control and long-term survival (10). Therefore, the risk of treatment-related symptoms might change with the advent of new treatment-combination modalities especially in real-world settings.

Immune checkpoint inhibitor-related pneumonitis is a challenging entity to diagnose, and currently there is no specific diagnostic test or symptoms which can outline ICIP patients. The most common scenarios with ICIP-like symptoms, such as infection and malignant lung infiltration, should always be initially ruled out. The risk of severe ICIP (grade 3 and 4) has been reported in randomized controlled trials more frequently in patients that receive monotherapy with CTLA-4 targeting agents (2−4%), followed by anti-PD-1 (1−3%) and anti-PD-L1 (0.4%) monoclonal antibodies (9, 11–13). Moreover, the rate increases (10%) when combinations with anti-PD-1/PD-L1 plus anti–CTLA-4 monoclonal antibodies are used (9), however, the incidence of ICIP may be underreported, as suggested by several retrospective studies of real-world data, in which the percentage of patients with ICIP is higher (10−20%) (14, 15).

The association with the history of radiotherapy and the risk of developing pneumonitis after treatment with ICIs has been scarcely described. We hypothesized that the risk of pneumonitis in patients that receive treatment with immunotherapy after radiotherapy increases compared with those who do not have a history of radiotherapy.

## Materials and Methods

### Study Design and Patient Selection

We performed a retrospective cohort study to evaluate the incidence of ICIP on patients with advanced NSCLC who had previously undergone radiotherapy and were currently receiving ICIs in the second-line setting, as well as its effect in terms of survival outcomes. Patients who were treated at the National Cancer Institute in Mexico City between February 2013 and February 2018 were screened for inclusion. Patient demographic, clinical and pathological characteristics, immunotherapy treatment type, previous history of radiotherapy, irradiated region and doses (radiation doses were considered additive among different regions), previous chemotherapy and other outcomes were collected from electronic medical records. Every medical charts and image studies were evaluated by a multi-disciplinary team including at least: a medical oncologist, thoracic surgeon and a radiation oncologist. Infectious processes were excluded by utilizing blood cultures or sputum cultures, as needed by each patient; furthermore, every patient received clinical evaluation by an experienced medical oncologist prior to every treatment session.

Patients treated concomitantly with immunotherapy and radiotherapy were excluded.

### ICIP Definition and Grading

All CT-scan were retrospectively reviewed. ICIP diagnosis was determined using a combination of clinical, radiological and biological tests to rule out differential diagnoses such as disease progression, infections, other comorbidities as well as radiation recall pneumonitis (pneumonitis limited to the radiation field). Pneumonitis toxicity grade was assessed according to the National Cancer Institute (NCI) Common Terminology Criteria for Adverse Events version 4.0 (CTCAE v4.0). Immune checkpoint inhibitors therapy was suspended in all patients who presented with G2 or higher ICIP and who required treatment with high doses of corticosteroids.

### Statistical Analysis

For descriptive purposes, continuous variables were summarized as arithmetic means and standard deviations (SDs), and categorical variables were expressed as frequencies and proportions. The χ2 and Fisher exact tests were used for evaluating the statistical significance between patient and treatment characteristics and the development of any grade of CIP. Progression free survival (PFS) was determined from the initiation of immunotherapy until progression of disease; OS was determined from initiation of immunotherapy until death by any cause or loss to follow-up. PFS and OS were analyzed by the Kaplan-Meier Method; risk factors for time to development of pneumonitis and univariate survival analysis were modeled by using a Cox proportional hazards model. All statistical tests were two-sided and *p* < 0.05 was deemed to be statistically significant. SPSS software (version 22; SPSS; Chicago, IL, United States) was used for data analysis.

## Results

A total of 101 NSCLC patients treated with immune checkpoint inhibitors as second line were included for the analysis. Median age for all population was 61 years old (±12.3). Most patients were female (57.4%) and had a positive smoking history (53.5%). The most common histological subtype was adenocarcinoma (84.1%). PD-L1 status was known in 35.6% of patients (36/101 patients), of whom, 75% (27/36 patients) were positive. Other baseline characteristics of the cohort are presented on Table 1.

**TABLE 1:** Demographic characteristics.

	Total (*n* = 101)
	*n* (%)
**Sex**	
Female	58 (57.4)
Male	43 (42.6)
**Age (years)**	
Mean (SD)	61.07 (±12.34)
<60 years	45 (44.6)
≥60 years	56 (55.4)
**History of smoking**	
Never	47 (46.5)
Smoker	54 (53.5)
**Woodsmoke exposure**	
No	78 (77.2)
Yes	23 (22.8)
**ECOG**	
0	10 (9.9)
1	88 (87.1)
≥2	3 (3)
**Stage**	
III	11 (10.9)
IV	90 (89.1)
**Histology**	
Adenocarcinoma	85 (84.1)
Squamous	11 (10.9)
Adenosquamous	5 (5)
**CNS Metastases**	
Yes	31 (30.7)
No	70 (69.3)
***EGFR* mutation**	
Positive	16 (15.8)
Negative	76 (75.2)
Undetermined	9 (8.9)
***ALK* mutation**	
Positive	0 (0)
Negative	88 (87.1)
Undetermined	13 (12.9)
***KRAS* mutation**	
Positive	0 (0)
Negative	34 (33.7)
Undetermined	67 (66.3)
**PDL-1 status**	
Positive	27 (26.7)
Negative	9 (8.9)
Undetermined	65 (64.4)
**First-line therapy**	
Platinum + Taxane	39 (38.6)
Platinum + Pemetrexed	34 (33.7)
Platinum + Gemcitabine	6 (5.9)
EGFR TKI	14 (13.9)
Other	8 (7.9)
**Immunotherapy**	
Nivolumab	42 (41.6)
Pembrolizumab	59 (58.4)
**Radiotherapy prior to ICI**	
Yes	40 (39.6)
No	61 (60.4)
**Radiotherapy dosage**	
<60 Gy	21 (52.5)
≥60 Gy	19 (47.5)

Regarding the treatment scheme, 41.6% (42/101) of patients were treated with nivolumab and 58.4% (59/101) with pembrolizumab as second-line of treatment. Among the included population, 40 patients (39.6%) received radiotherapy prior to ICI therapy; additionally, among radiotherapy-treated patients 17 (42.5%) received radiotherapy exclusively to the lung, 20 (50%) received radiotherapy to the vertebral column and three (7.5%) to mediastinal lymph nodes. The overall incidence of any-grade ICIP was 21.8% (22/101 patients). Incidence of ICIP in patients with history of radiotherapy was significantly higher compared with radiotherapy-naïve patients [40% vs. 9.8%; OR 6.11; 95% CI 2.13**−**17.52 (*p* < 0.001)]. In addition, doses greater than 60 Gy of radiation were associated with an increased risk of developing ICIP (OR 7.21; 95% CI 1.83**−**28.40) compared to patients who received less than 60-Gy (OR 5.35; 95% 1.56**−**18.42), however, this was not statistically significant.

Median time from ICI initiation to pneumonitis onset was 4.5 months (range 0.72**−**13.14 months). No association was found between line of treatment and the elapse time to ICIP development. The incidence of ICIP was similar between both ICI drugs (54.5% vs. 45.5% for nivolumab and pembrolizumab, respectively, *p* = 0.16).

**FIGURE 1:**
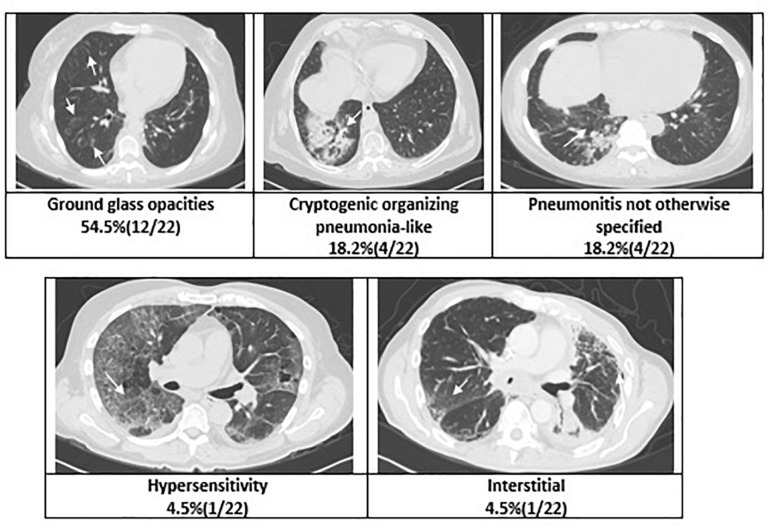
Radiographic patterns of pneumonitis.

Grade ≥ 2 ICIP developed in 12 patients (11.9%); and grade ≥ 3 in four patients (4%). Incidence of grade ≥ 2 ICIP was also higher in patients who received previous radiotherapy (22.5% vs. 4.9%). Remarkably, all patients that developed grade ≥ 3 pneumonitis had been previously treated with radiotherapy (Table 2). Despite the fact that tomography patterns can be superimposed, predominantly ground glass opacities, we can classify the damage based on the predominant injury; the tomographic pattern more frequently found was ground glass opacities, which was seen in 50% (12/22 patients), cryptogenic organizing pneumonia-like and pneumonitis not otherwise specified were found in 18.2% (4/22 patients), besides interstitial lung pattern, and hypersensitivity pneumonitis were reported (4.5% in both cases) (Figure 1).

**TABLE 2:** Characteristics among patients who experienced ICIP.

	Pneumonitis (Any Grade)			Pneumonitis (Grade ≥ 2)			Pneumonitis (Grade ≥ 3)		
	No pneumonitis	Pneumonitis	*p*	No pneumonitis	Pneumonitis	*p*	No pneumonitis	Pneumonitis	*p*
**All patients (%)**	79 (78.2)	22 (21.8)		89 (88.1)	12 (11.9)		97 (96)	4 (4)	
**Sex**									
Female	45 (77.6)	13 (22.4)	**0.85**	52 (89.7)	6 (10.3)	**0.57**	57 (98.3)	1 (1.7)	**0.20**
Male	34 (79.1)	9 (20.9)		37 (86)	6 (14)		40 (93)	3 (7)	
**Age (years)**									
<60 years	38 (84.4)	7 (15.6)	**0.17**	42 (93.3)	3 (6.7)	**0.14**	44 (97.8)	1 (2.2)	**0.39**
≥60 years	41 (73.2)	15 (26.8)		47 (83.9)	9 (16.1)		53 (94.6)	3 (5.4)	
**History of smoking**									
Never	34 (72.3)	13 (27.7)	**0.18**	40 (85.1)	7 (14.9)	**0.38**	45 (95.7)	2 (4.3)	**0.63**
Smoker	45 (83.3)	9 (16.7)		49 (90.7)	5 (9.3)		52 (96.3)	2 (3.7)	
**Woodsmoke Exposure**									
No	66 (86.8)	10 (13.2)	**0.02**	71 (93.4)	5 (6.6)	**0.01**	74 (97.4)	2 (2.6)	**0.19**
Yes	13 (56.5)	10 (43.5)		17 (73.9)	6 (26.1)		21 (91.3)	2 (8.7)	
**ECOG**									
<2	79 (80.6)	19 (19.4)	**0.01**	88 (89.8)	10 (10.2)	**0.01**	94 (95.9)	4 (4.1)	**0.88**
≥2	0 (0)	3 (100)		1 (33.3)	2 (66.7)		3 (100)	0 (0)	
**Stage**									
III	7 (63.6)	4 (36.4)	**0.21**	9 (81.8)	2 (18.2)	**0.49**	11 (100)	0 (0)	**0.62**
IV	72 (80)	18 (20)		80 (88.9)	10 (11.1)		86 (95.6)	4 (4.4)	
**Histology**									
Adenocarcinoma	69 (81.2)	16 (18.8)	**0.24**	76 (89.4)	9 (10.6)	**0.64**	82 (96.5)	3 (3.5)	**0.6**
Squamous	7 (63.6)	4 (36.4)		9 (81.8)	2 (18.2)		10 (90.9)	1 (9.1)	
Adenosquamous	3 (60)	2 (40)		4 (80)	1 (20)		5 (100)	0 (0)	
***EGFR* mutation**									
Positive	13 (81.3)	3 (18.8)	**0.66**	13 (81.3)	3 (18.8)	**0.65**	16 (100)	0 (0)	**0.39**
Negative	58 (76.3)	18 (23.7)		68 (89.5)	8 (10.5)		73 (96.1)	3 (3.9)	
Undetermined	8 (80)	1 (20)		8 (88.9)	1 (21.1)		8 (88.9)	1 (11.1)	
**PDL-1 status**									
Positive	19 (70.4)	8 (29.6)	**0.17**	22 (81.5)	5 (18.5)	**0.29**	26 (92.9)	2 (7.1)	**0.53**
Negative	9 (100)	0 (0)		9 (100)	0 (0)		10 (100)	0 (0)	
Undetermined	51 (78.5)	14 (21.5)		58 (89.2)	7 (10.8)		61 (96.8)	2 (3.2)	
**Chemotherapy**									
Platinum + Taxane	29 (85.3)	10 (25.6)	**0.36**	34 (87.2)	5 (12.8)	**0.82**	38 (97.4)	1 (2.6)	**0.77**
Platinum + Pemetrexed	29 (85.3)	5 (14.7)		31 (91.2)	3 (8.8)		33 (97.1)	1 (2.9)	
Platinum + Gemcitabine	4 (66.7)	2 (33.3)		5 (83.3)	1 (16.7)		6 (100)	0 (0)	
**Prior TKI treatment**									
No	65 (76.5)	20 (23.5)	**0.44**	75 (88.2)	10 (11.8)	**0.79**	81 (95.3)	4 (4.7)	**0.40**
Yes	12 (85.7)	2 (14.3)		12 (85.7)	2 (14.3)		14 (100)	0 (0)	
**Immunotherapy Drug**									
Nivolumab	30 (71.4)	12 (28.6)	**0.16**	34 (81)	8 (19)	**0.6**	40 (95.2)	2 (4.8)	**0.72**
Pembrolizumab	49 (83.1)	10 (21.8)		55 (93.2)	4 (6.8)		57 (96.6)	2 (3.4)	
**Radiotherapy**									
Yes	24 (60)	16 (40)	**0.01**	31 (77.5)	9 (22.5)	**0.01**	36 (90)	4 (10)	**0.01**
No	55 (90.2)	6 (9.8)		58 (95.1)	3 (4.9)		61 (100)	0 (0)	

*Bold text indicate analyzed characteristics and corresponding p values.*

**FIGURE 2:**
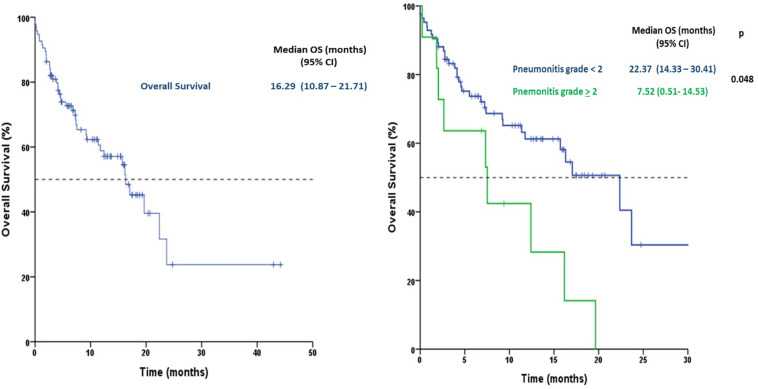
Kaplan-Meyer curves for the association between checkpoint-immune pneumonitis and mortality.

Median PFS was 3.6 months (95% CI 2.6**−**4.6) and median OS was 16.3 months (95% CI 10.9**−**21.7). There were no significant differences in OS between patients who developed ICIP or those who did not. However, developing grade ≥ 2 pneumonitis conferred the patients a statistically significant increased risk in mortality (HR 2.54, 95% CI 1.20**−**5.34, *p* = 0.014), Figure 2.

Mean follow-up was of 9.22 months (range 0.22 – 44.2). Follow up was performed by scheduling medical visits ever 3-weeks. Patients that did not presented to scheduled visits during 2 months were contacted by a phone call; those patients that were unable to be contacted were censored from analysis.

In addition, a one, three, and six-months landmark analysis confirmed a trend toward a better OS at every point for the patients without ≥ grade 2 pneumonitis; with landmark analysis reaching statistical significance at 6 months [median OS of 23.7 (95% CI NR-NR) months for patients without pneumonitis ≥ grade 2 vs. 16.2 months (95% CI 6.8 – 25.6) for patients with pneumonitis ≥ grade 2; p 0.047]; landmark analysis at 1 month almost reached statistical significance for better OS in patients without pneumonitis ≥ grade 2 when compared with patients that developed pneumonitis ≥ grade 2 [median OS 22.4 months (95% CI 14.9 – 29.8) vs. 12.4 months (95% CI 5.5 – 19.4); *p* = 0.057], (Supplementary Figure 1).

A wide univariate analysis was performed to evaluate many base-line and treatment characteristics (Table 3). Furthermore, clinically relevant variables, and those that reached statistical significance at the univariate analysis were analyzed and adjusted through a multivariate model which is also presented at Table 3.

**TABLE 3:** Factors associated with overall survival.

				Unadjusted		Adjusted	
	No. (Events)	Median (95% CI)	*p*-Value	HR (95% CI)	*p*-Value	HR (95% CI)	*p*-Value
**Overall**	95 (42)	16.29 (10.8−21.7)					
**Sex**							
Female	55 (20)	19.6 (15.3−23.9)					
Male	40 (22)	12.4 (10.6−21.4)	0.18	0.66 (0.36−1.22)	0.18	1.56 (0.34−1.18)	0.15
**Age (years)**							
<60	42 (21)	16.2 (10.6−21.9)					
≥60	53 (21)	19.6 (5.3−33.9)	0.62	0.85 (0.46−1.57)	0.62	0.81 (0.44−1.50)	0.51
**Histology**							
Adenocarcinoma	81 (35)	16.3 (12.9−19.7)					
Other	14 (7)	7.3 (NA-NA)	0.64	1.21 (0.53−2.74)	0.64		
**Tobacco exposure**							
No	46 (19)	16.2 (10.0−22.5)					
Yes	49 (23)	17.0 (5.5−28.5)	0.72	0.94 (0.69−1.28)	0.72		
**Wood-smoke exposure**							
No	72 (33)	16.1 (11.1−21.1)					
Yes	23 (29)	19.9 (2.68−36)	0.46	0.75 (0.36−1.59)	0.45		
**ECOG PS**							
<2	92 (39)	17.0 (10.9−23.1)					
≥2	3 (3)	16.1 (2.01−30.30)	0.67	1.11 (0.47−2.64)	0.80		
**CNS Metastases**							
No	65 (29)	16.2 (10.7−21.6)					
Yes	30 (13)	18.2 (10.9−21.7)	0.99	0.99 (0.52−1.92)	0.98		
***EGFR* mutation**							
Absent (wild-type)	71 (29)	17.0 (13.2−20.8)					
Present (*EGFR* mutated)	15 (8)	12.4 (5.8−1.2)	0.21	1.24 (0.18−1.89)	0.3		
**Radiotherapy**							
Yes	40 (19)	17.5 (10.4−23.7)					
No	55 (23)	16.2 (8.4−24.0)	0.930	0.97 (0.52−1.80)	0.93		
**Pneumonitis**							
No	74 (31)	16.2 (9.5−23.0)					
Yes	22 (11)	16.1 (10.3−22.0)	0.71	1.13 (0.57−2.26)	0.71		
**Pneumonitis >2**							
No	84 (33)	22.3 (14.3−30.41)					
Yes	12 (9)	7.5 (0.51−14.5)	**0.048**	2.48 (1.18−5.23)	**0.028**	2.54 (1.20−5.34)	**0.014**

*Bold text indicate analyzed characteristics and corresponding p values.*

## Discussion

Lung toxicity presents with low frequency, but is often a serious treatment treatment-related complication in patients with NSCLC receiving ICI therapy. We retrospectively analyzed the frequency of ICIP and its correlation with prior radiotherapy in a cohort of patients with advanced NSCLC from a single medical center. Our results show that ICIP incidence in the real world scenario might be higher than reported in clinical trials (5, 7), with an all-grade ICIP incidence of 21.8% and a grade ≥ 3 ICIP of 4%, however, the frequency of ICIP increased when patients had a history of radiotherapy (40%).

Several studies have shown that the presence of irAEs is associated with a higher efficacy and improved overall survival in several solid tumors treated with ICIs, including NSCLC (16–18). Nonetheless, ICIP might be an exemption to this rule. As suggested by the publication of Suresh K. et al., ICIP increases mortality in patients with NSCLC, this observation is reported in patients with adenocarcinoma subtype histology, in whom risk of death increased proportionally with ICIP grade. These previous results support the findings in this study. Overall our data suggests that a history of radiation therapy increases the risk of ICIP, and increases mortality.

We observed an increased ICIP rate among patients with prior thoracic radiation, which supports the immunomodulatory effect of radiotherapy that converts an entirely or partially non-immunogenic tumor into an immunogenic one. Cell-death induced by radiotherapy generates molecular signals and inflammatory cytokines that promote the ability of dendritic cells to release antigens to T cells (19).

Different clinical trials have proven the benefit of using combination therapy. The PACIFIC trial demonstrated the advantage of using combined treatment modalities with chemo-radiation and anti-PD-L1 (durvalumab) compared with chemo-radiation and placebo in patients with NSCLC. PFS was 16.8 months in the durvalumab group versus 5.6 months in the placebo group also with a higher response rate (28.4% vs. 16% *p* < 0.001). However, the combination treatment leads to an increased risk of any grade of pneumonitis as observed in both groups (33.9% vs. 24.8%), as well as represents the most frequent adverse event leading to treatment discontinuation. A secondary analysis from phase 1 KEYNOTE-001 evaluated disease control and pulmonary toxicity in 97 patients with NSCLC that received pembrolizumab (20). This analysis reported that 8% (2/24) of patients who received prior chest-radiotherapy developed ICIP, compared with 1% (1/73) of patients who had not received prior chest-radiotherapy. Similarly, our results show that the incidence of ICIP was higher in the sample of patients who received prior radiotherapy. The KEYNOTE sub analysis also showed that OS was longer in patients who received pembrolizumab and radiotherapy, and in those who received extracranial radiotherapy, compared with those who did not (HR 0.59 95% CI 0.36**−**0.96). This supports the possibility of an enhancing the effect of radiotherapy on the immune system by combining this with pembrolizumab. Patients with a history of thoracic radiation had an overall higher frequency of treatment-related pulmonary toxicity in 63% (15/24) compared with no-previous lung radiotherapy with 40% (29/73) *p* = 0.052 (20).

The ongoing PEMBRO-RT trial was randomized phase II study that evaluated the improvement in overall response rate (ORR) at 12 weeks in 76 patients with NSCLC receiving pembrolizumab with or without prior stereotactic body radiotherapy (SBRT). The ORR at 12 weeks was 18% in the control arm vs. 36% in the experimental arm (*P* = 0.07). Median progression-free survival was 1.9 months (95% CI, 1.7**−**6.9 months) vs. 6.6 months (95% CI, 4.0**−**14.6 months) (hazard ratio, 0.71; 95% CI, 0.42**−**1.18; *P* = 0.19), and median overall survival was 7.6 months (95% CI, 6.0**−**13.9 months) vs. 15.9 months (95% CI, 7.1 months to not reached) (hazard ratio, 0.66; 95% CI, 0.37**−**1.18; *P* = 0.16), (21).

Furthermore, patients who previously received thoracic radiotherapy were more likely to have any grade of pulmonary toxicity. These data highlight the role of radiotherapy in priming the immune response and thereby potentiating immune-mediated toxicity, also known as radiation recall syndrome. Radiation recall pneumonitis (RRP) is a specific subtype of radiation pneumonitis that occurs after the trigger of cytotoxic agents in a previous radiated lung. While the exact mechanism of RRP development is not yet well understood, the clinical, radiological and functional characteristics of the patients are similar as in radiation pneumonitis and the diagnosis is based on the premise of a previously radiated lung and the exposure to a triggering agent. The time range of RRP appearance since the triggering agent completion is wide (22**−**169 days) with a mean of 47 days (22). A diverse range of chemotherapeutic agents have been related to RRP, while only a few associations with molecular-targeted therapy have been reported (23).

As such, in this study we build on the previous evidence regarding the incidence of ICIP in NSCLC patients treated with ICIs and a history of radiation. The strength of this association is considerable (OR 6.04), however, another previous study which reported 15 cases of ICIP among ICI treated patients had also identified a strong relationship between ICIP incidence and previous radiotherapy history. In this study, the authors report that 67% of patients with ICIP had undergone radiotherapy, nonetheless, the study included patients with a wide variety of neoplasms, including melanoma, esophageal cancer and lung cancer, encompassing thus a heterogeneous sample. Despite this observation, the authors highlight that lung regions affected by the primary tumor, metastasis or radiotherapy had a significantly higher probability of ICIP compared with others, with an OR of 10.8 (24).

The relative high risk of pneumonitis among NSCLC patients may be explained because they are prone to develop drug-related lung toxic effects associated with several factors inherently related to the patient demographics, including their exposure to tobacco and underlying lung conditions (chronic obstructive pulmonary disease and pulmonary fibrosis). Existing tumor burden in the lung may also limit the lung tolerance to exogenous stress and injury. These underlying conditions may contribute to more serious clinical consequences from lung injury during pneumonitis. Drug-induced pneumonitis remains an exclusion diagnosis, and requires consideration of competing diagnoses, including infection and disease progression.

As it has been noted, there are huge differences among reported incidence of ICIP in patients with NSCLC that receive immunotherapy; the exact reason of such huge variations is not known, however, it might be related to ethnicity of studied population, awareness and recognition of ICPI, and study design. Lack of and specific diagnostic test for ICPI further complicates its diagnosis and reporting system. Therefore, we consider that it is important to evaluate the incidence and risk factors for ICIP in a real-world population with a future prospective study. In the immunotherapy era, the anti-cancer therapy has been extended the reach of the immune system and many findings are extremely encouraging. However, the adverse effects of the novel combination therapies should be considered. Improvements in the treatment and understanding of the biology of pneumonitis are needed to optimize and maximize the therapeutic effect of checkpoint inhibitors in NSCLC patients.

## Conclusion

Results from this study show that ICIP incidence is higher in real-world settings compared with clinical trials, with up to 21.8% of patients treated with ICIs diagnosed with ICIP. Our data might suggest that a prior history of radiotherapy could increase the risk of developing ICIP, which in turn might increase the mortality. However, prospective studies are needed to corroborate our results as well as to appropriately identify the true incidence and risk factors for developing ICIP. Beside this, the appropriate dose and modality of radiotherapy, and the concomitant or sequential use of immunotherapy should be explored in clinical trials.

## Data Availability Statement

All datasets presented in this study are included in the article/Supplementary Material.

## Ethics Statement

Ethical review and approval was not required for the study on human participants in accordance with the local legislation and institutional requirements. Written informed consent for participation was not required for this study in accordance with the national legislation and the institutional requirements.

## Author Contributions

FB, RS, and OA: conception or design of the work. FB, RS, MA-H, CB, MR-R, and DF-E: data collection. FB, RS, and RC: data analysis and interpretation. RS, ZZ-B, and RC: drafting the article. FB, AC, and OA: critical revision of the article. All authors approved the final version to be published.

## Conflict of Interest

OA reports personal fees from Pfizer, grants and personal fees from AstraZeneca and Boehringer Ingelheim, personal fees from Lilly, Merck, and Bristol Myers Squibb, grants and personal fees from Roche, outside the submitted work. The remaining authors declare that the research was conducted in the absence of any commercial or financial relationships that could be construed as a potential conflict of interest.
