# Correction: Risk of developing checkpoint immune pneumonitis and its effect on overall survival in non-small cell lung cancer patients previously treated with radiotherapy

**DOI:** 10.3389/fonc.2026.1783059

**Published:** 2026-02-06

**Authors:** Feliciano Barrón, Roberto Sánchez, Marisol Arroyo-Hernández, Carolina Blanco, Zyanya L. Zatarain-Barrón, Rodrigo Catalán, Maritza Ramos-Ramírez, Andrés F. Cardona, Diana Flores-Estrada, Oscar Arrieta

**Affiliations:** 1 Thoracic Oncology Unit, Instituto Nacional de Cancerología, México City, Mexico; 2 Cancer Center, ABC Medical Center, México City, Mexico; 3 Clinical and Translational Oncology Group, Clínica del Country, Bogotá, Colombia; 4 Foundation for Clinical and Applied Cancer Research – FICMAC, Bogotá, Colombia; 5 Clinical Research and Biology Systems Department, Universidad el Bosque, Bogotá, Colombia

**Keywords:** checkpoint immune therapy, pneumonitis, radiotherapy, NSCLC, lung cancer, immune related adverse effects

In the published article, the “No. (Events)” values for the “Wood-smoke exposure” row and Pneumonitis rows in **Table 3** were transcribed incorrectly during table formatting. The Cox analysis for wood-smoke exposure and both pneumonitis categories (any grade and ≥2) were performed with the correct underlying data; only the “No. (Events)” figures in that row are misprinted.

**Table 3:** Factors associated with overall survival.

				Unadjusted		Adjusted	
	No. (Events)	Median (95% CI)	p-Value	HR (95% CI)	p-Value	HR (95% CI)	p-Value
**Overall**	95(42)	16.29 (10.8-21.7)					
Sex							
Female	55(20)	19.6(15.3-23.9)					
Male	40(22)	12.4(10.6-21.4)	0.18	0.66 (0.36-1.22)	0.18	1.56 (0.34-1.18)	0.15
Age (years)							
<60	42(21)	16.2(10.6-21.9)					
≥60	53(21)	19.6(5.3-33.9)	0.62	0.85 (0.46-1.57)	0.62	0.81 (0.44-1.50)	0.51
Histology							
Adenocarcinoma	81 (35)	16.3 (12.9-19.7)					
Other	14 (7)	7.3 (NA-NA)	0.64	1.21 (0.53-2.74)	0.64		
Tobacco exposure							
No	46(19)	16.2(10.0-22.5)					
Yes	49(23)	17.0(5.5-28.5)	0.72	0.94 (0.69-1.28)	0.72		
Wood-smoke exposure							
No	72(33)	16.1(11.1-21.1)					
Yes	23(29)	19.9(2.68-36)	0.46	0.75 (0.36-1.59)	0.45		
ECOG PS							
<2	92(39)	17.0(10.9-23.1)					
≥2	3(3)	16.1(2.01-30.30)	0.67	1.11 (0.47-2.64)	0.80		
CNS Metastases							
No	65 (29)	16.2 (10-7-21.6)					
Yes	30 (13)	18.2 (10.9-21.7)	0.99	0.99 (.52-1.92)	0.98		
*EGFR* mutation							
Absent (wild-type)	71(29)	17.0 (13.2-20.8)					
Present (*EGFR* mutated)	15(8)	12.4 (5.8-1.2)	0.21	1.24 (0.18-1.89)	0.3		
Radiotherapy							
Yes	40(19)	17.5(10.4-23.7)					
No	55(23)	16.2(8.4-24.0)	0.930	0.97 (0.52-1.80)	0.93		
Pneumonitis							
No	73(31)	16.2(9.5-23.0)					
Yes	22(11)	16.1(10.3-22.0)	0.71	1.13 (0.57-2.26)	0.71		
Pneumonitis >2							
No	83(33)	22.3(14.3-30.41)					
Yes	12(9)	7.5(0.51-14.5)	** 0.048**	2.48 (1.18-5.23)	**0.028 **	2.54 (1.20-5.34)	**0.014**

Bold text indicate analyzed characteristics and corresponding p values.

The corrected table appears below.

The original version of this article has been updated.

