# Supplementary material for: Risk of Developing Checkpoint Immune Pneumonitis and Its Effect on Overall Survival in Non-small Cell Lung Cancer Patients Previously Treated With Radiotherapy
Source: Front Oncol. 2020 Sep 29;10:570233. doi: 10.3389/fonc.2020.570233 (PMC7550759; doi:10.3389/fonc.2020.570233)

Supplementary Figure 1. Landmark analysis for overall survival at 1months (S. Figure 1-A), 3 months (S. Figure 1-B) and 6 months (S. Figure 1-C) in patients without grade ≥ 2 pneumonitis vs patients with grade ≥ 2 pneumonitis

Supplementary Figure 1-A. Landmark analysis for OS at 1 month

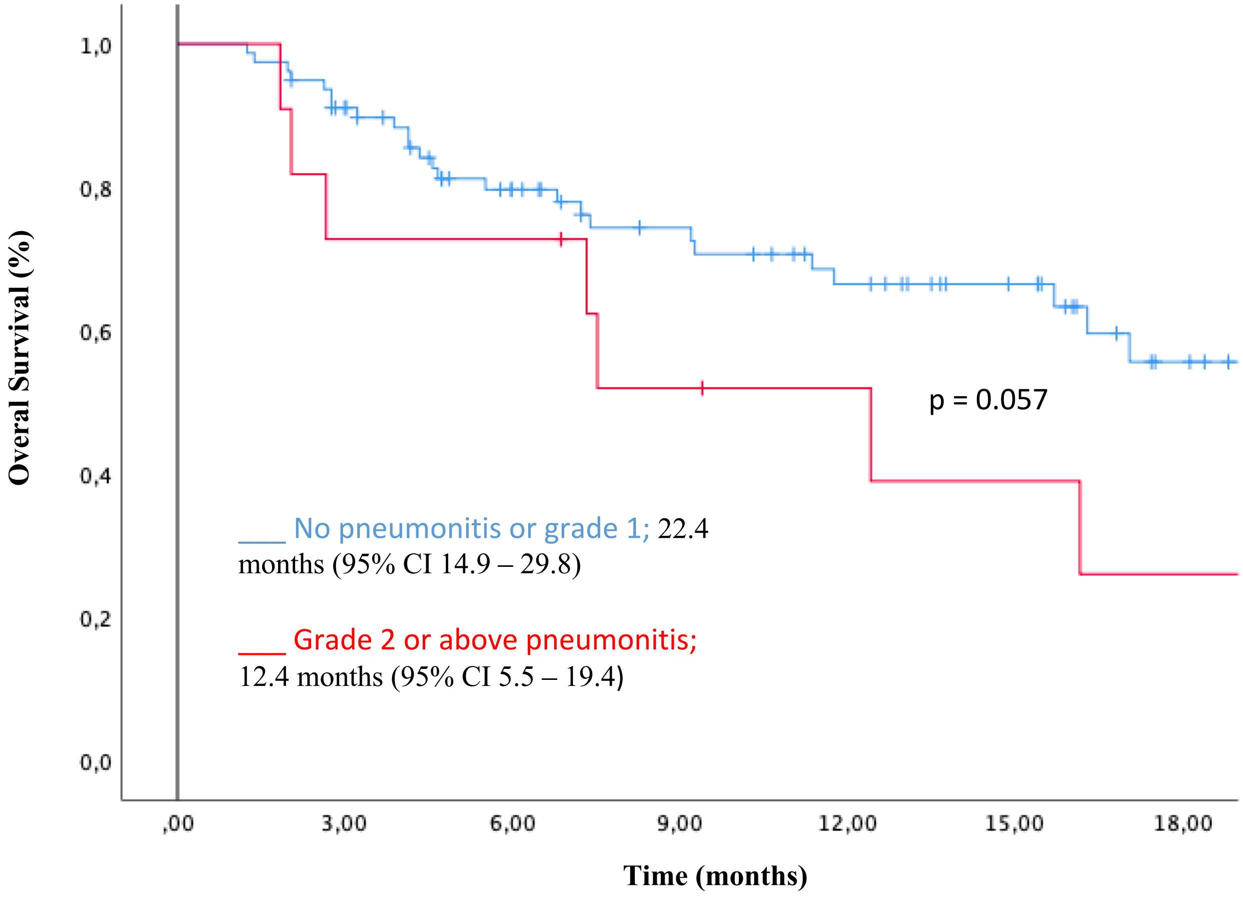

Supplementary Figure 1-B. Landmark analysis for OS at 3 months

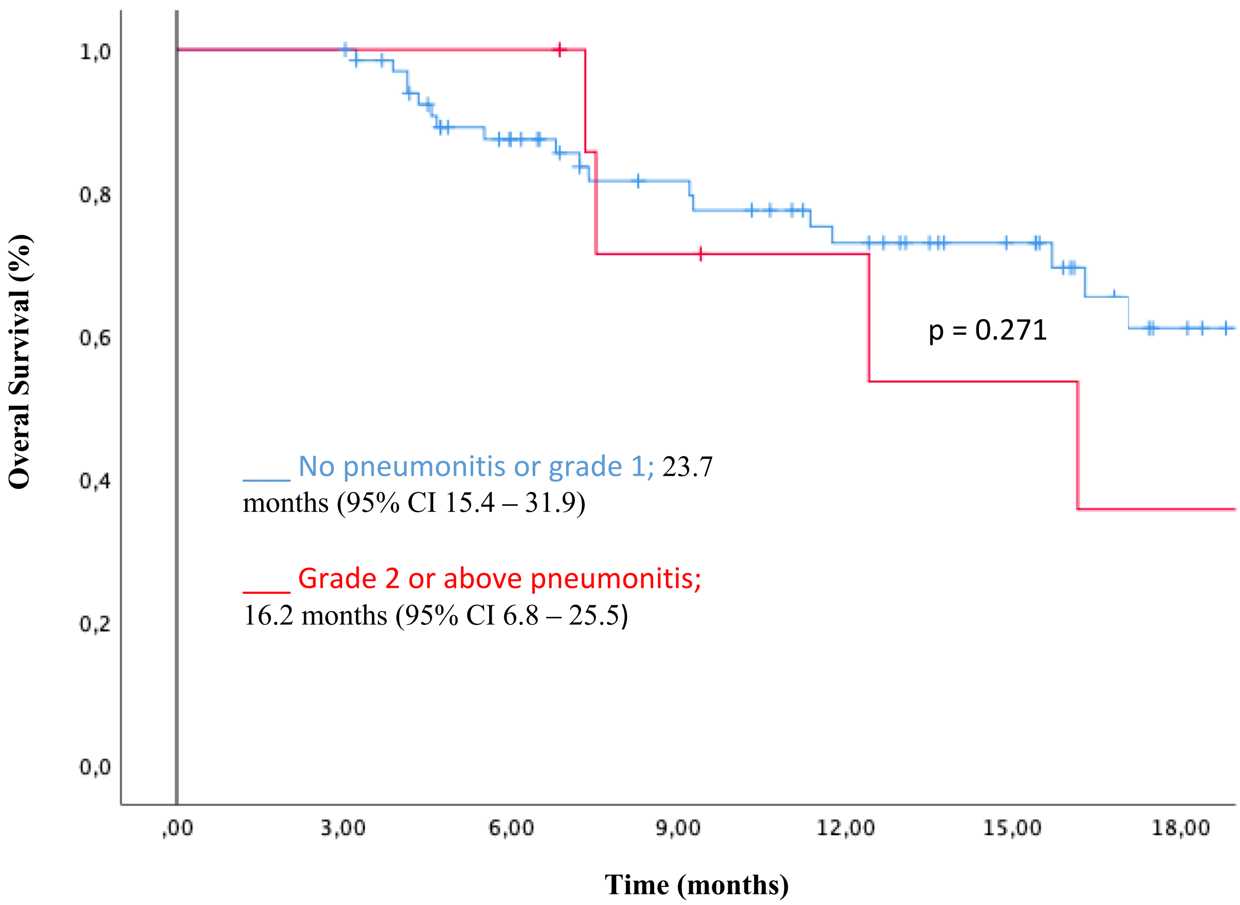

Supplementary Figure 1-C. Landmark analysis for OS at 6 months

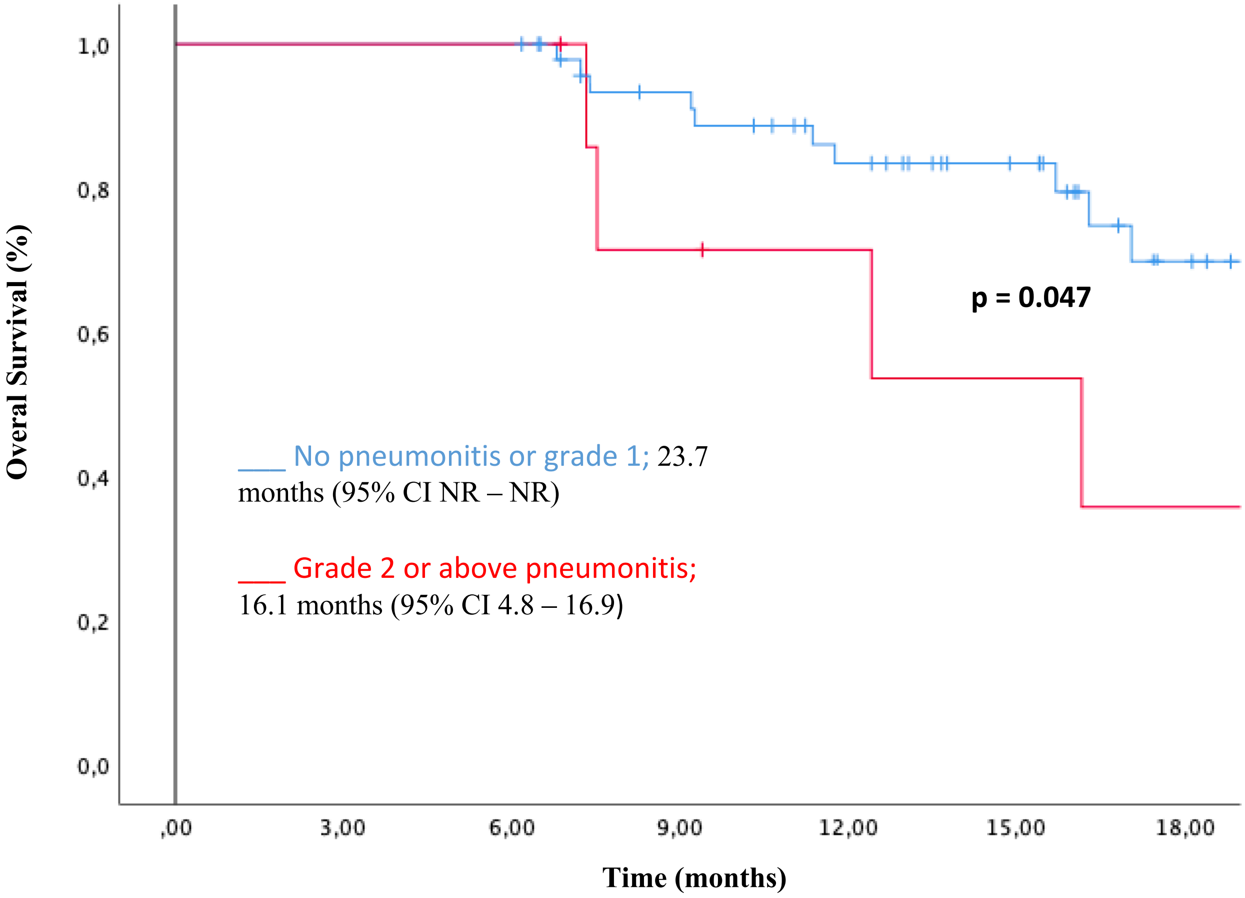

Supplement: Supplementary file 1 [file Image_1.PDF]
